# Using system dynamics modelling to assess the economic efficiency of innovations in the public sector - a systematic review

**DOI:** 10.1371/journal.pone.0263299

**Published:** 2022-02-10

**Authors:** Nidhee Jadeja, Nina J. Zhu, Reda M. Lebcir, Franco Sassi, Alison Holmes, Raheelah Ahmad

**Affiliations:** 1 NIHR Health Protection Research Unit in Healthcare Associated Infections and Antimicrobial Resistance at Imperial College London, Imperial College London, London, United Kingdom; 2 University of Hertfordshire Business School, Hatfield, United Kingdom; 3 Department of Economics & Public Policy, Centre for Health Economics & Policy Innovation, Imperial College Business School, South Kensington Campus, London, United Kingdom; 4 Division of Health Services Research and Management, School of Health Sciences, City, University of London, London, United Kingdom; Wageningen University, NETHERLANDS

## Abstract

**Background:**

Decision-makers for public policy are increasingly utilising systems approaches such as system dynamics (SD) modelling, which test alternative interventions or policies for their potential impact while accounting for complexity. These approaches, however, have not consistently included an economic efficiency analysis dimension. This systematic review aims to examine how, and in what ways, system dynamics modelling approaches incorporate economic efficiency analyses to inform decision-making on innovations (improvements in products, services, or processes) in the public sector, with a particular interest in health.

**Methods and findings:**

Relevant studies (n = 29) were identified through a systematic search and screening of four electronic databases and backward citation search, and analysed for key characteristics and themes related to the analytical methods applied. Economic efficiency analysis approaches within SD broadly fell into two categories: as embedded sub-models or as cost calculations based on the outputs of the SD model. Embdedded sub-models within a dynamic SD framework can reveal a clear allocation of costs and benefits to periods of time, whereas cost calculations based on the SD model outputs can be useful for high-level resource allocation decisions.

**Conclusions:**

This systematic review reveals that SD modelling is not currently used to its full potential to evaluate the technical or allocative efficiency of public sector innovations, particularly in health. The limited reporting on the experience or methodological challenges of applying allocated efficiency analyses with SD, particularly with dynamic embedded models, hampers common learning lessons to draw from and build on. Further application and comprehensive reporting of this approach would be welcome to develop the methodology further.

## 1. Introduction

Complex global problems such as climate change or antimicrobial resistance need innovation to shape impactful policies, systems, and services. As the current Covid-19 crisis reveals, the public sector plays a critical role in steering change to tackle the world’s most wicked problems; it is the only actor with the necessary legitimacy and resources to do so [1]. The world’s public sectors face acute fiscal and effectiveness pressures to tackle major challenges, it is therefore essential to ensure that policies represent good value for money. Innovation in the public sector refers to the implementation of a new or significantly changed product, which could be a good, service, or process, which can include production or delivery, organisation, and marketing processes [2]. Ex-ante simulation modelling of innovations in the health sector can help guide decision-makers, providing insight into how scenarios of different public sector innovations might play out in real-world settings. The notion of innovation has positive connotations attached to it, but a simulation model can reveal whether it creates any desired impacts or even possibly deleterious ones.

### 1.1. Using system dynamics to model complex public sector problems

Systems science approaches are increasingly being used to shape public sector innovations as they recognise [3, 4] the complexity of systems and mitigate the limitations of reductionist analytic modelling methods used to analyse these problems. Gault (2018) makes the case for a systems approach to analysing innovation in the public sector, recognising the potentially far-reaching impacts of actions beyond one specific sector and as a basis for developing more comprehensive policies [2]. In 2006 the United States Biomedical Advanced Research and Development Authority (BARDA) utilised a public health systems science approach to plan for pandemic influenza [5]. System Dynamics (SD) modelling is one such systems science approach that was originally developed in management science to represent and explain complex behaviours in a system such as patterns of non-linearity, externalities, and counterintuitive outcomes [6]. It uses computer simulation models to help address problems in complex systems and test alternative policies and scenarios in a systematic way [7]. SD tools such as causal loop diagrams (CLDs) and stock-flow diagrams (SFDs), are used to capture the non-linear mechanisms of a complex system [8]. These diagrammatic tools map the feedback structures and show how the system is dynamically influenced by the interactions of all variables [9].

Within the suite of systems methodologies, SD offers additional capabilities for informing intervention design and policy-making in comparison to soft systems methodologies by integrating qualitative and quantitative elements to represent soft behavioural variables, and engaging decision-makers in the process of testing policies or intervention strategies based on real-world circumstances [5, 10]. Qualitative approaches, including interviews and focus groups, can help elucidate key causal influences and factors in responding to a problem [11]. The participatory approach to model building in SD which engages stakeholders throughout the process ensures that real-world circumstances are taken into account. It enables organizational learning, aims to align stakeholder understanding of the underlying cause of and potential solutions to a problem, and facilitates consensus on the course of action and eventual policy adoption [12, 13]. SD has been gaining importance in informing health sector innovations as it can address common challenges in traditional approaches to policy-making, such as policy resistance, where actions triggered as a result of a policy undermine the policy or even exacerbate the original problem [14–17]. Public sector resources however are finite and it is unclear how SD modelling has incorporated economic efficiency analyses, which provides crucial insight for policymakers in their decision-making.

### 1.2. The value of economic efficiency analyses for decision-making

Economists usually distinguish between two types of efficiency: technical and allocative efficiency. Technical efficiency refers to maximising activities or outcomes from a fixed set of resources, while allocative efficiency is concerned with directing resources to their most productive use to achieve the best overall benefits [18, 19]. Economic efficiency analyses, such as cost-effectiveness or cost-utility studies, compare options by their resource needs and subsequent benefits [20]. Economic efficiency analyses meaningfully contribute to health sector decisions by helping to set priorities and cost-effective plans, identifying the best ways of achieving strategic objectives, and providing insight on returns on investment [21]. Economic efficiency evaluations are an established practice in helping to inform public health sector decisions, however, on their own they typically represent a static snapshot of the situation rather than the shifting cost and benefit dynamics in the system over time [22, 23].

### 1.3. Aim and objectives

The aim of this systematic review was to examine and describe the range and nature of economic efficiency analyses in SD studies to understand how the shifting cost and benefit dynamics in the system have been evaluated for public sector innovations for complex problems. There was a particular interest for this review in the health sector, given the complex nature of health sector challenges and need for efficient use of resources. The specific objectives were to 1) Determine the policy target level (macro, meso, or micro level) for which the analysis has been conducted, 2) Compare approaches for how economic efficiency analyses have been incorporated with SD, and 3) Evaluate the quality and completeness of reporting of the economic efficiency analyses and SD modelling using the Consolidated Health Economic Evaluation Reporting Standards (CHEERS) checklist and Rahmandad and Sterman’s guidelines for reporting for simulation-based research in social sciences [24, 25].

## 2. Method

A systematic search was conducted to identify articles published from 1st January 1999 to 1^st^ June 2021 from the *Scopus*, *Medline*, *EMBASE*, *Web of Science* and *Econlit* databases using the search terms indicated in S1 Table. All the databases were last searched on the 10^th^ June 2021. The review was limited to the past 22 years so as to reflect recent developments and current applications of SD. For both *Medline* and *EMBASE*, both keywords and MeSH terms were used. To further identify relevant articles, backward citation searches of two recent systematic reviews of SD modelling and health were conducted [17, 26, 27]. Only papers in English were included. Papers eligible for inclusion were those that described applications of an economic efficiency analysis in SD modelling to support a public sector decision-making process at any level of government and in any sector, to gain insights into the methodological approach itself. Studies excluded at both the title and abstract screening and full text screening were conference proceedings, those that did not use SD to assess the allocative efficiency between two or more policy options for a public sector, and those that were not available in the public domain. An example of a study that might appear to have met the inclusion criteria, but which was excluded is the study by Fontoura et al., which evaluated the impact of the existing Brazilian Urban Mobility Policy (BUMP) in the urban transport system, but did not involve an economic allocative efficiency analysis with SD to compare between two or more policy options [28]. Another example is the study by Lam and Mercure, which analyses which policy mixes are best for decarbonising passenger cars across five countries, assessing both the policies’ effectiveness in achieving emissions reductions and their cost-effectiveness in doing so [29]. This study was excluded as it did use System Dynamics modelling. Two reviewers (NJ and NJZ) independently screened all the study titles and abstracts and a third reviewer (CA) independently screened a randomised sample of 25% of the records using the software platform Covidence. Disparities were resolved through discussion to reach consensus. Following screening, two reviewers (NJ and NJZ) independently screened the full text of all manuscripts for inclusion into the review. The third reviewer (CA) independently screened a random sample of 25% of the studies. The detailed assessment of included studies was initially performed by one author (NJ) and reviewed by another author (RA). Once again, disparities were resolved through discussion and consensus. The reporting of this systematic review is in line with the Preferred Reporting Items for Systematic Reviews and Meta-Analyses (PRISMA) guidelines (please see S2 Table for checklist) [30].

The quality and completeness of reporting of the economic efficiency analyses and SD modelling were examined in order to identify key themes related to the specific analysis methods applied, type of public sector innovation, and limitations associated with the approach. Although the specific economic efficiency analyses and overall model objectives of the studies vary significantly given the differing sectors, there are well-accepted guidelines for good modelling practices that the studies can be assessed against. The Consolidated Health Economic Evaluation Reporting Standards (CHEERS) checklist, which provides guidance on good reporting practices in health economic evaluations, was adapted to assess the quality of reporting of the economic efficiency evaluations [24]. While the CHEERS checklist was developed for health economics evaluations specifically, it is largely applicable to economic evaluations more broadly, and thus was suitable as a benchmark/quality standard for which to assess the studies against. It includes analysis criteria such as target level of decision-making for policy, reporting of analytical methods, type of intervention or policy, limitations, and data sources. The quality and completeness of reporting of the SD model was assessed using Rahmandad and Sterman’s guidelines for reporting for simulation-based research in social sciences, which were developed to address the general lack of reporting guidelines in simulation-based research in the social sciences to facilitate the reproducibility of the simulation models [25]. The guidelines distinguish between factors essential for the reproduction of research and those that practice transparency, and only relevant guidelines were adapted and applied for this analysis and incorporated into the synthesis table referred to above. These include general visualisation guidelines, model reporting requirements, and simulation experiment reporting. Rahmandad and Sterman’s guidelines are more process-oriented and the CHEERS checklist is more focused on outcomes reporting; in this way they are a complementary set of criteria against which to evaluate the completeness of reporting of the studies.

The studies were also classified according to Windrum’s conceptualization of the six different types of public sector innovations, to understand where SD and economic evaluation has been applied [31]:

1. Services innovation–described as “new or altered service features and design”
2. Service delivery innovation–described as “new or altered ways of delivering services or interacting with citizens”
3. Administrative or organisational innovation–described as “new or altered organizational methods in public sector practices, workplace organization or external relations, increasing public sector’s performance by reducing administrative/transaction costs, improve workplace satisfaction, etc.”
4. Conceptual innovation–described as “the development of new world views that challenge assumptions that underpin existing service products, processes and organizational forms”
5. Policy innovation–described as “to change the thought or behavioural intentions associated with a policy/new or altered missions, objectives, strategies and rationales”
6. Systemic innovation–described as “new or improved ways of interacting with other organizations and knowledge bases”

The review protocol, template data collection forms, and data extracted are available upon request.

## 3. Results

### 3.1. Study selection

**Fig 1:**
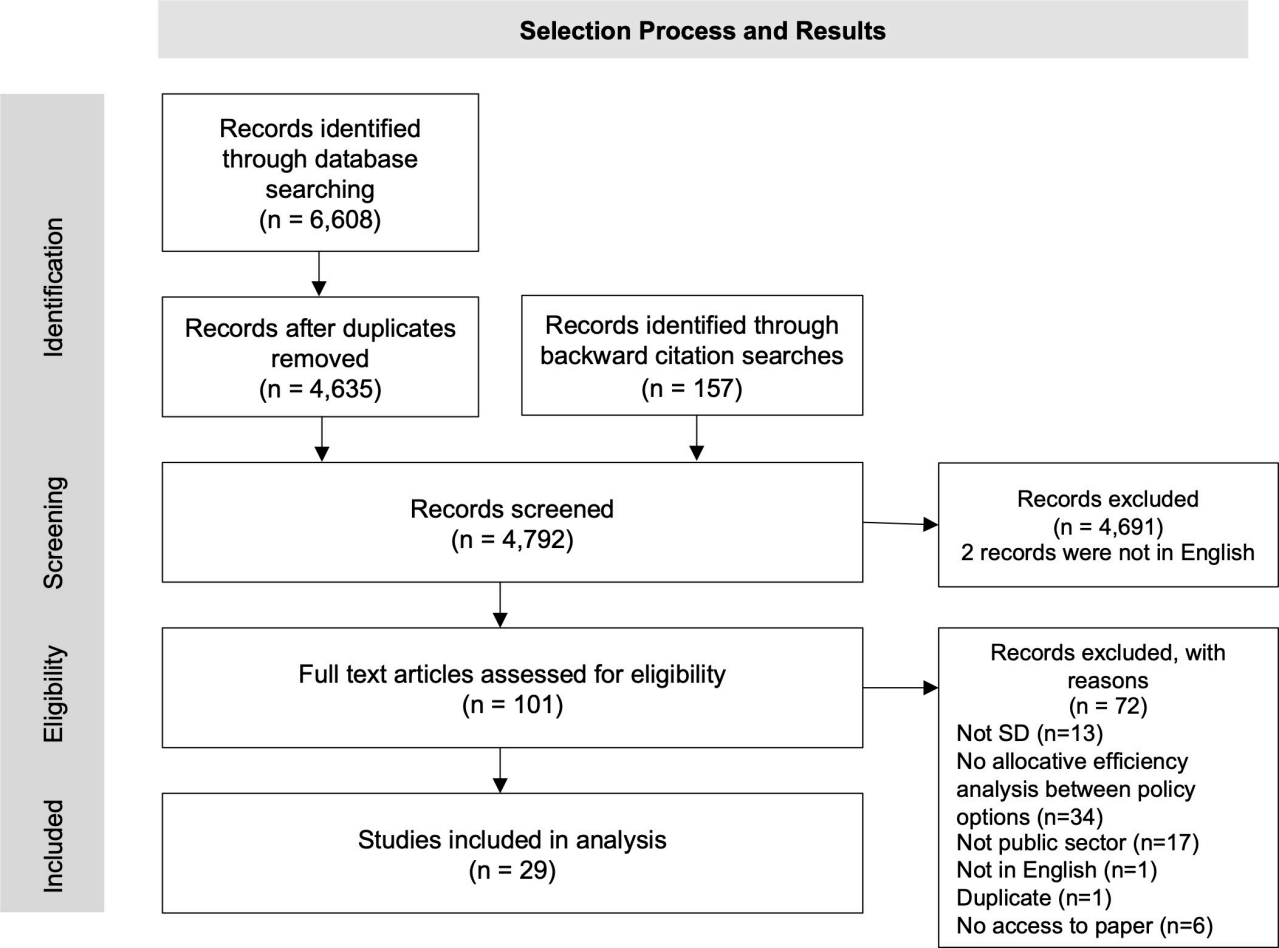
Selection process and results.

Fig 1 below summarises and describes the screening and checking process for final analysis and review. In total 6,608 records were identified through searching the databases. After removing duplicates and conducting backward citation searches, 4, 792 titles and abstracts were screened. From those screened, 101 full text articles were screened for eligibility and 29 studies were finally included for analysis.

### 3.2. General characteristics

Table 1 provides a summary of the general characteristics of the selected studies. Most of the studies (*n = 19*) were conducted in the latter half (2011–2021 of the review’s twenty-two year time period, which suggests an increasing need and recognition of SD to evaluate the cost aspects of public sector innovations. All the studies conducted a systematic analysis of the problem, and then used simulation to model to test the impacts of various innovation options.

**Table 1:** General characteristics of selected studies.

Sector	Author, year	Setting	Study objective	Public Sector Innovation Type and Target Level	Economic Efficiency Analysis & Finding
Health	Ahmad, 2009	California, USA	To evaluate of the cost-effectiveness of raising California state’s legal smoking age to 21.	Policy Innovation, Meso-level	**Cost-effectiveness analysis.** The policy would generate no net costs, saving $24 billion over 50 years with a gain of 1.47 million QALYs compared to status quo.
	Ahmad, 2005	USA	To estimate how a national law raising the smoking age to 21 would impact smoking prevalence, net costs and health benefits to the population over time.	Policy Innovation, Macro-level	**Cost-effectiveness analysis** The policy would produce a net cumulative savings to society of US$ 212 billion (driven by reduced medical costs), and the accumulation of nearly 13 million additional QALYs over the period.
	Ansah et al., 2021	Singapore	To project cost for dialysis for chronic kidney diseases (CKD) and end stage renal diseases (ESRD) and assess cost saving through upstream and downstream interventions.	Mix of service delivery innovation and policy innovation, Macro-level	**Cost / cost-saving analysis.** Findings support the current policy of promoting the use of peritoneal dialysis and expanding subsidized haemodialysis capacity, while simultaneously strengthening upstream prevention of CKD and ESRD, resulting overall in significant cost savings over time.
	Duintjer Tebbens and Thompson, 2009	National	To investigate how changes in perceptions of priorities might play out in the context of multiple eradicable diseases in a hypothetical population competing for resources. The study evaluates policies that focus resources on the disease perceived as having the highest incidence at any particular time versus policies that pursue eradication.	Policy innovation, macro level	**Incremental Cost-effectiveness Ratio** The analysis shows that the eradication policies yield better incremental cost-effectiveness ratios than control policies, and the need to sustain commitment to eradication even when the perceived urgency of the disease declines.
	Erten et al., 2016.	Vermont Medical Centre, USA	To compare the costs and ascertainment of targeted versus universal screening of Colorectal cancers for Lynch syndrome	Service delivery innovation, micro level	**Total costs and costs saved** Targeted screening costs 2- to 7.5-fold less than universal and rarely misses Lynch syndrome cases.
	Evenden et al., 2005	Portsmouth, UK	To capture Chlamydia infection dynamics and conduct a cost-benefit study for screening.	Service delivery innovation, micro level	**Cost-benefit analysis** Screening provides immediate cost benefits, and to achieve optimal cost savings, a larger proportion of the high-risk groups need to be screened.
	Evenden et al, 2020	UK	To assess cost-utility for lifestyle interventions to delay the onset of dementia.	Policy Innovation, Macro-level	**Cost-utility analysis** QALY gained through lifestyle intervention compared with medication (also measured cost saving per patient)
	Honeycutt et al, 2019	USA	To assess community-based tobacco control interventions	Mix of service delivery innovation and policy innovation, macro level	**Cost-effectiveness analysis** $735 million in medical costs and 3750 deaths could be averted from 2010 through 2020. The interventions would remain cost saving even if maintenance costs were incurred, with incremental cost effectiveness from cost saving to $239,300 per death averted.
	Kivuti-Bitock et al., 2014	Kenya	To evaluate the possible effect of primary vaccination, secondary vaccination and screening campaigns for Kenya in the area of Cervical Cancer Management.	Service Delivery innovation, Macro-level	**Cost-utility analysis** The simulation results demonstrate that an increase in the level of coverage of the different interventions resulted in an increase in the reduction of DALYs as well as an increase in DALYs averted.
	Hirsh et al., 2014	USA	To explore how 4 distinct categories of interventions differ in terms of their potential for reducing the risks of cardiovascular disease (CVD) in a population over a 30-year time horizon.	Mix of service delivery innovation and policy innovation, Macro-level	**Total costs** Taxes and regulation reduce costs the most in the short term and long term and reduce deaths the most in the long term; they are second to clinical interventions in reducing deaths in the short term.
	Hirsch et al., 2012	Colorado, USA	To determine which interventions, singly or in combination, could have the greatest effect in reducing caries experience and cost in a population of children from birth to 5 years.	Mix of service delivery innovation and policy innovation, meso level	**Total costs and costs saved** Interventions targeting the highest-risk children provide the highest return on investment.
	Homer et al., 2010	USA	To evaluate multiple approaches to preventing and managing cardiovascular risks, in terms of first-time cardiovascular events, consequent deaths, as well as total consequence costs.	Mix of service delivery innovation and policy innovation, macro level	**Total costs and costs saved** At least 15 of 19 interventions are potentially cost saving and could reduce deaths from first cardiovascular events by approximately 20% and total consequence costs by 26%.
	Mahmoudian-Dehkordi, et al., 2017	Intensive Care Unit	To estimate the long-term effects of expanding ICU versus IMCU beds on patient lives.	Services innovation, Micro-level	**Incremental Cost-effectiveness Ratio** Based on the ICER of dollars per life saved, ICU expansion is superior to introducing IMCU.
	Milstein et al., 2011	USA	To evaluate three proposed large-scale intervention strategies for reducing deaths and improve the cost-effectiveness of interventions.	Mix of service delivery innovation, policy innovation, and systemic innovation, Macro level	**Cost-effectiveness analysis** Expanding health insurance coverage and delivering better preventive and chronic care save lives quickly but tend to increase costs. The impact of protecting health by enabling healthier behaviour and improving environmental conditions grows more gradually but lowers deaths and costs over time.
	Sluijs et al, 2021	Netherlands	To develop an SD model for policy makers and health professionals to gain a clear understanding of the patient journey of type 2 diabetes mellitus and to assess the impact of lifestyle intervention programs on total cost for society associated with prevention and lifestyle treatment of pre-diabetes and type 2 diabetes in The Netherlands.	Mix of service delivery innovation and policy innovation, and systemic innovation, Macro level	**Cost-saving / cost-benefit analysis** The model shows that the integrated program and integrated personalised care program prove to be most effective in terms of long-term societal cost reduction, and no intervention is the least effective one.
	Smith and Van Ackere, 2002	UK	To show how it has become possible to integrate conventional micro-economic models into the SD framework in order to provide readily accessible guidance to decision-makers on the dynamic implications of economic models.	Organizational innovation, Macro-level	**Total costs** The paper demonstrated how it is possible to embed a simple static economic model within a dynamic framework using SD.
	Tejada et al, 2013	USA	To develop and exploit a two-phase simulation modelling framework for evaluating the effectiveness of screening and treatment of breast cancer in the growing population of U.S. women who are at least 65 years of age.	Service delivery innovation, Macro-level	**Cost-effectiveness analysis** Annual breast cancer screening for all women 65–80 maximises lives saved and minimises the cost per QALY saved.
	Tengs et al., 2001	USA	To evaluate the short- and long-term costs, health gains, and cost-effectiveness of delivering an intensive school-based tobacco use prevention program to every 7^th^ and 8^th^ grade student in the United States.	Services innovation, Macro-level	**Cost-effectiveness analysis** More intensive school-based anti-tobacco educational efforts would be economically efficient investments.
	Tuulonen et al., 2009	Finland	To test and rank different options for access to eye care and the required physician workforce.	Mix of services innovation and service delivery innovation, Macro-level	**Total public sector costs** Specific initiatives on price level of new technologies, treatments, and practice patterns will be important to restrain healthcare costs efficiently.
	Yarnoff et al, 2019	USA	To use the Prevention Impacts Simulation Model, a SD model of CVD prevention, to simulate the potential impact of clinical and community interventions implemented by 32 communities receiving a Community Transformation Grant program award.	Policy Innovation, Macro-level	**Cost effectiveness analysis** The 10-year cost effectiveness of clinical activities was $302,000 per premature death prevented and $169,000 per premature death prevented for community activities. The 25-year effectiveness of clinical activities was $188,000 per premature death averted, and the 25-year effectiveness of community activities was $57,000 per premature death averted.
Climate Change	Alirezaei et al., 2017	USA	To model the climate change-road safety-economy nexus, thereby investigating the complex interactions among these.	Policy innovation, Macro-level	**Economic impact on GDP** Reducing GHG emissions and improving the vehicle safety index can significantly reduce road accident fatalities and thereby result in economic benefits.
Water	Assaf, 2009	Jordan	To assess three aquifer depletion and water allocation policies over a period of 50 years.	Policy innovation, Meso-level	**Return on investment** Agricultural water economic return is very low in comparison to municipal and industrial counterparts. A no-depletion policy produces the highest economic return. Higher discount rates values may significantly undervalue water conservation measures.
	Chen, 2020	Taiwan	To assess cost saving by greenhouse gas emission through water saving policies.	Policy Innovation, Macro-level	**Cost-saving / cost-benefit analysis (cost per ton greenhouse gas emission reduced)** The environmental cost of GHG emissions associated with water use behaviour was US$0.001/t, causing an 8% cost increase, which was acceptable to the respondents in this study.
	Van Zyl et al, 2020	Cape Town, South Africa	A system dynamics model of Cape Town’s water system serves as a case study to evaluate policy interventions, aimed at extracting value from retainable and recyclable water sources to address the growing water shortage experienced in cities.	Policy Innovation, Macro-level	**Cost-saving / cost-benefit analysis** Greywater systems in produce more than six times the amount of water for reuse, in comparison to decentralised wastewater treatment plants, albeit at a much higher cost.
Transport	Al-Foraih, 2020	Bangladesh	To evaluate of the economic benefits and associated environmental gains of under three scenarios (replacing private vehicles with public transport facilities).	Policy Innovation, Macro-level	**Cost / cost-saving analysis.** By replacing 70% private vehicles with public buses, economic savings can be achieved through reduced fuelling cost.
	Macmillan et al., 2014	Auckland,New Zealand	To develop a commuter cycling and public health model integrating physical, social, and environmental well-being to identify cost-effective transport policies for improving public health.	Mix of conceptual innovation and policy innovation, Meso-level	**Cost-benefit analysis** Best practice physical separation on main roads and bicycle-friendly speed reduction on local streets would yield benefits 10–25 times greater than costs.
	Schade and Rothengatter, 2005	European Union	To develop an SD model that allows for a dynamic CBA integrating the most important indirect effects of transport policies.	Policy innovation, Macro-level	**Cost-benefit analysis** The choice of the most favourable policy can change over time and depend on the time horizon defined for analysis.
Energy	Shih and Tseng, 2013	Taiwan	To conduct a cost-benefit analysis of the economic feasibility of the Sustainable Energy Policy Guidelines for climate change mitigation	Mix of conceptual innovation and policy innovation, Macro-level	**Cost-benefit analysis** Renewable Energy has higher benefit-cost ratios than Energy Efficiency Improvements.
Housing	MacAskill et al, 2021	Australia	To explore how a recent shift towards bond-based funding mechanisms offer an opportunity to integrate green building practices, and influence social outcomes.	Policy Innovation, Macro-level	**Costs/Cost-benefit analysis.** The Green building framework would deliver 2.37% less housing overall due to higher initial capital costs however, it would offer substantial long-term benefits and efficiency.

### 3.3. Policy target level and geography

**Fig 2:**
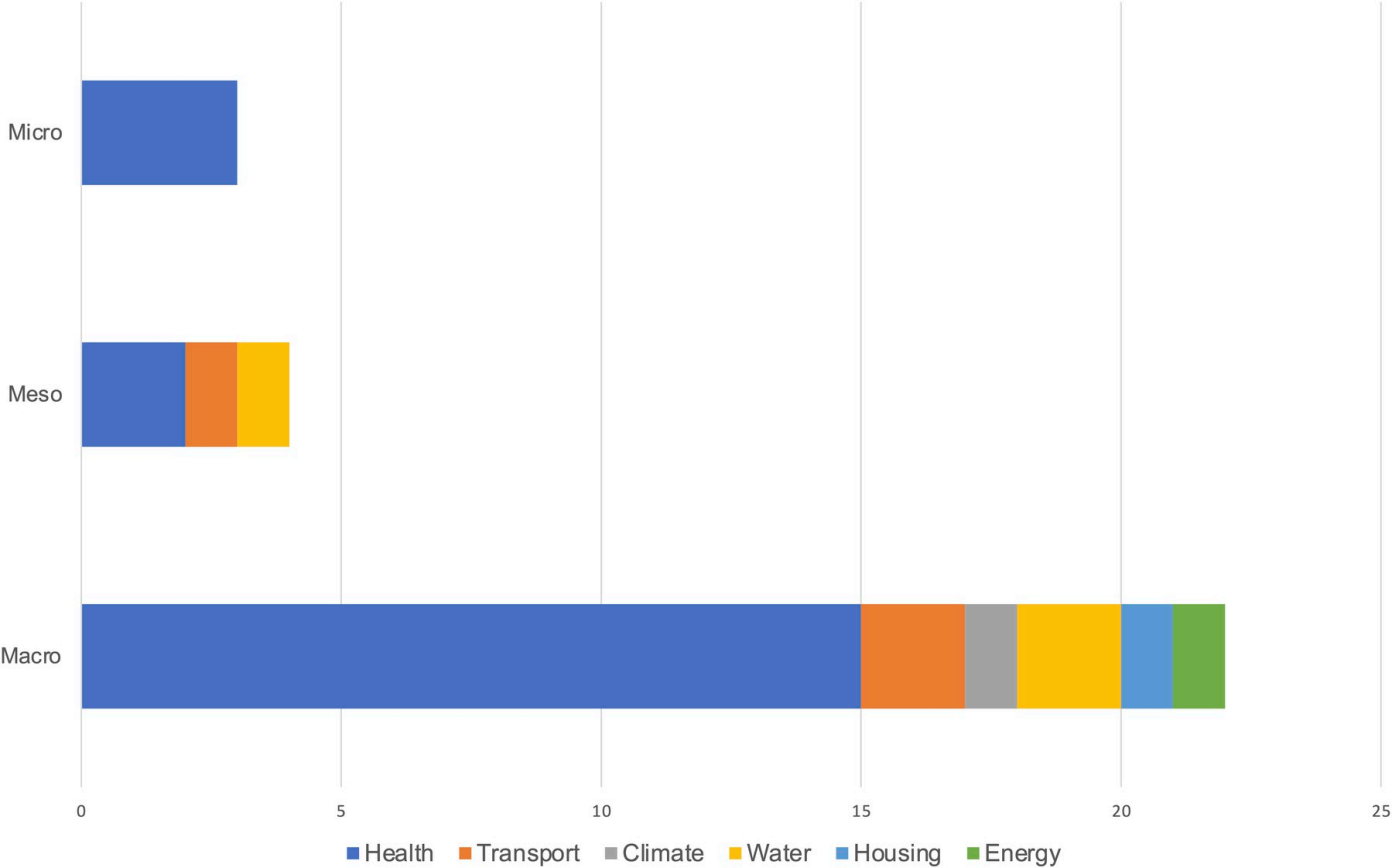
Number of studies by policy target level.

One of the aspects examined was the target level of the public sector innovation, which was classified as either macro (national), meso (regional, municipal), or micro (hospital, ICU unit) level (Fig 2). Seventy-six percent of the studies addressed innovations at the macro level, and four studies addressed innovations at the meso level. Ahmad et al. evaluated the policy of raising the legal smoking age at both the macro (i.e. in the USA) and meso (regional, i.e. in California) level through two separate published analyses, as tobacco control policies can be mandated at both the national and state levels in the USA [32, 33]. Three studies used economic efficiency analysis with SD to inform decision-making at the micro level. Mahmoudian-Dehkordi and Sadat (2017), for example, compared intensive care units (ICU) versus step-down or intermediate care unit (IMCU) capacity expansion in hospitals [34]. In terms of geography, twelve of the study settings were focused on the USA, with four studies focused on a low- and middle-income country setting.

### 3.4. Type of public sector and innovation

**Fig 3:**
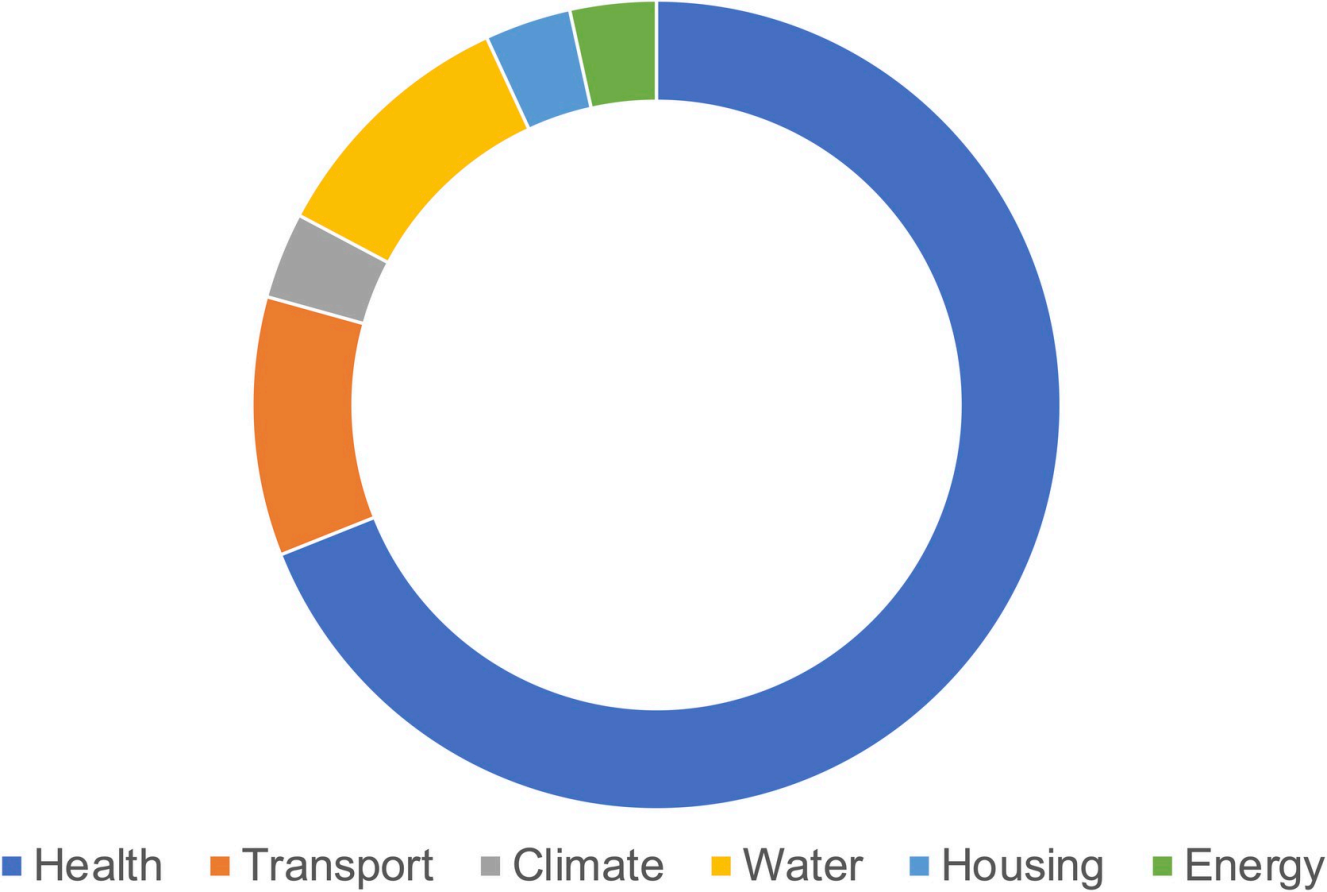
Number of studies by type of public sector.

The studies used SD to inform decision-making across a number of different sectors, which included health (*n = 20*), transport (*n = 3*), climate (*n = 1*), water (*n = 3)*, housing (*n = 1)*, and energy (*n = 1*) sectors (Fig 3). Within health, the innovations being evaluated focused on both infectious and non-communicable diseases (NCDs), with Kivuti-Bitock’s evaluation of HPV vaccination and cervical cancer screening interventions in Kenya spanning both [35]. Countries around the world are facing increasing populations affected by ageing-associated diseases and conditions, and many of the studies in this review in the health sector examined the question decision-makers face regarding the balance between chronic disease prevention and management strategies. For example, Ansah et al. explored the health impact, costs, and cost savings of upstream and downstream interventions on the future number of chronic kidney disease and dialysis care patients in Singapore by 2040 [36]. Similarly, Sluijs et al. developed an SD model for policy-makers to understand and assess the impact and cost-effectiveness of lifestyle intervention programs on type 2 diabetes in the Netherlands [37]. A heterogenous mix of public sector innovation types were evaluated across the studies. According to Windrum’s typology, twenty studies evaluated policy innovations. These included a raising of the legal smoking age [32], reductions in CO2 emissions [38, 39], and regulations on groundwater aquifer use [40]. All of the studies evaluating services innovation (n = 2) or service delivery innovation (n = 11) or both (n = 1) were in the health sector. These included expanded provision for beds in intensive care units [34], increasing access to eye care services [41], screening and treatment services for breast cancer [42], and more intensive school-based anti-tobacco educational efforts [43].

Six of the studies sought to produce or use a generic or hypothetical simulation model in a specific public sector for future use by decision-makers. Alirezaei et al. for example, used SD to understand the complex interdependencies of the climate change-road safety-economy nexus itself, and develop a model platform that can be subsequently used by policymakers, rather than generate results of the model itself [38]. Duintjer Tebbens and Thompson model a hypothetical population in which two eradicable infectious diseases circulate, and evaluate different policy decision options on addressing them to show that cost-effectiveness decreases as the extent of priority-shifting increases [23]. Their study reveals how unintended consequences can arise from what might be considered intuitive decision rules in infectious disease control, and highlights the need to assess the costs and benefits of different policies when making decisions related to complex systems [*ibid*]. Three studies used a SD simulation model named The *Prevention Impacts Simulation Model* (PRISM) to explore the impacts of different interventions aimed at reducing cardiovascular disease. PRISM is a system dynamics model, originally developed in 2005 and funded by the US Centers for Disease Control and Prevention (CDC) and National Institutes of Health National Heart, Lung, and Blood Institute (NIH NHLBI), to simulate the health and cost outcomes for the entire US population from 1990 to 2040 and analyse the potential impacts of strategies to address cardiovascular disease risk factors [44]. The model reports summary measures of mortality and years of life lost and the medical and productivity costs of the chronic diseases and has been used by decision-makers at the local and federal level (*ibid*). Hirsch et al. used PRISM to explore the multiyear impacts of 22 different interventions aimed at reducing cardiovascular disease [45]. Yarnoff et al. used PRISM to simulate the potential 10-year and 25-year impact of clinical versus community interventions implemented by 32 communities in the United States, revealing the trade-offs decision-makers have to grapple with–clinical interventions had the potential to avert more premature deaths than community interventions, however, community interventions sustained over the long-term were more cost-effective [46]. Finally, Honeycutt et al. used PRISM to examine the potential cost-effectiveness of tobacco control changes implemented under a CDC-funded programme across 50 communities in the United States [47].

Other studies assessed specific innovations and compared the results to provide recommendations to decision-makers. Tejada et al., for example, compose discrete-event simulation (DES) and SD sub-models to evaluate the effectiveness of new or altered service delivery options for the screening and treatment of breast cancer in women 65+ in the USA [42]. Erten et al. compared targeted versus universal screening of colorectal cancers for Lynch Syndrome in terms of diagnostic accuracy and cost differences using real-world clinical data and not hypothetical assumptions [48].

### 3.5. Type of economic efficiency analysis

As shown in Table 1, the types of economic efficiency analyses conducted across the included studies were examined to better understand the capability of SD to incorporate different types. The most common type within the studies was cost-effectiveness analyses (CEA) of the innovations (*n = 9*), though the specific approaches to this varied. For example, Tejada et al. calculated the cost effectiveness ratio “average cost per quality adjusted life years (QALY) saved” in their breast cancer screening-and-treatment simulation for a ten year period using existing cost and QALY data from the US Department of Health and Human Services and academic literature [42]. Two studies conducted a cost-utility analysis (CUA). Kivuti-Bitock et al (2014) conducted a CUA, evaluating the effect of primary vaccination, secondary vaccination and screening campaigns for cervical cancer management in Kenya [35]. Evenden et al. developed a hybrid simulation model for dementia care services planning, showing that the currently available interventions of medication and a healthy lifestyle have only modest effects in terms of QALYs and reduced costs at the population level [49]. Disability Adjusted Life Years (DALYs), which represent the loss of the equivalent of one year of full health, and cost per averted DALY were used in the cost utility analysis [50]. DALYs consisted of Years of Life Lost (YLL) and Years of Life Lived with Disability (YLD), and the cost per averted DALY was based on a simplified calculation based on the total cost of intervention divided by the DALYs averted. Eight studies across the health, transport, water, housing, and energy sectors included cost-benefit analyses (CBA) [37, 51–57]. MacMillan et al., for example, compared the effects of policy innovations to increase bicycle commuting in Auckland through a participatory SD approach. To ensure the policy relevance of their findings, they applied the New Zealand national transport agency’s methods to calculate indicative benefit-cost ratios for each policy scenario compared with baseline (summed net benefits divided by infrastructure costs) [51]. Evenden et al. developed a SD model for capturing Chlamydia infection dynamics within a population, and provide a cost-benefit study for required screening rates to manage infection prevalence [54].

Ten studies simply sought to assess the total cost impact of the innovations in question, though in these cases it was very important to understand how ‘cost’ was defined. Hirsch et al. for example, considered the effects of the interventions on deaths and downstream (or ‘consequence’) costs, with ‘costs’ referring to discounted (at 3% per year) direct medical costs for risk factor management and preventive care, acute care for CVD events and other risk factor-related hospitalizations, post-CVD long-term care, as well as productivity costs due to disability from CVD events and premature deaths from CVD events and other risk factor complications [45]. The costs did not include the administrative or non-medical implementation costs of interventions. In another study evaluating interventions for early childhood caries, Hirsch et al. report on both cumulative costs of restorative care and program costs as well as savings in restorative care compared to no intervention [58]. Data from dental offices and ambulatory or hospital sites was obtained to calculate the cost savings attributable to avoided restorative care from various interventions [*ibid*.]. Homer et al. avoided attempting to quantify intervention costs, which can be more difficult to estimate for broad classes of interventions through diverse strategies, and instead focused on consequence costs, arguing that this can still valuably help guide decision-makers by serving as a benchmark to justify the costs of interventions [59]. For example, if a given intervention results in a total consequence cost saving of $50 per capita, decision-makers can then justifiably spend up to $50 per capita for a given intervention and still maintain a net positive benefit [*ibid*.]. Consequence costs and savings were measured by medical and productivity (morbidity and mortality) costs using a human capital approach which estimates the market value of lost productivity at work and at home [*ibid*.].

CEA, which calculates a cost per unit of outcome for each intervention, and cost utility analysis (CUA), in which the incremental cost of an intervention or innovation is compared to the incremental benefit, are the most common forms of economic evaluation in health [60, 61]. Cost-effectiveness and cost-utility studies provide the investment case for choosing one innovation over another, but the focus on a single outcome can often limit its ability to capture the comprehensive range of costs and benefits [62]. CBA in contrast, synthesises and valuates all costs and benefits of an innovation in monetary units, and allows for a broader range of outcomes in monetary terms [61]. In theory this should allow for greater ease of comparison across innovations, but it is considered more vulnerable to bias in decision-making, as the included costs and benefits have to be measurable [60]. While none of the studies provided a rationale or justification for their selected approach, the different economic evaluation approaches conducted demonstrates the capability of SD to accommodate a wide range of types.

This review found that economic efficiency analysis approaches with SD within these studies broadly fell into two categories: as embedded sub-models or as cost calculations based on the outputs of the SD model. Smith and Ackere were motivated by the fact that decision-makers are often interested not just in the equilibrium predictions arising from an economic model, but also in the path taken by policy variables as they move towards equilibrium [63]. They demonstrate how it is possible to embed a simple static economic model within a dynamic SD framework, using the NHS as an example, to enhance the usefulness of the economic model [*ibid*]. Schade and Rothengatter take the rationale a step further, arguing that alternative approaches to traditional static models are needed for cost-benefit analyses [52]. They developed an SD platform that integrates a dynamic CBA of transport policies, revealing that the most favourable policy can change over time and depend on the time horizon defined for the analysis [*ibid*]. Their approach allows for a clear allocation of costs and benefits to periods of time, which may be particularly valuable for facilitating policy acceptance and implementation [*ibid*]. Milstein et al. also provide this type of dynamic temporal insight in their study of how the US system responds to large-scale interventions [64]. They demonstrate that while expanded health insurance coverage and better preventive and chronic care can save lives quickly, they tend to increase costs, and it is improved health behaviour and environmental conditions which are the critical ingredient over time for lowering both the number of deaths and reducing costs [*ibid*]. Ahmad on the other hand, used a SD simulation model to estimate smoking prevalence rates from policy changes to the legal smoking age, and then applied calculations of economic impacts to these outputs in terms of medical cost savings, cost of law enforcement, and cost of checking identification [32, 33].

### 3.6. Quality and completeness of reporting

Table 2 summarises the completeness of reporting of the economic efficiency analysis and SD modelling according to the CHEERS checklist and Rahmandad and Sterman’s guidelines in the selected studies, with a checkmark indicating where relevant information was provided. As can be seen, eighteen studies reported limitations and challenges however all of them related to the assumptions and estimations, rather than the technical aspects or application of economic efficiency analysis with SD or even to SD itself. The limited reporting on the experience or methodological challenges of applying cost analyses with SD, particularly with dynamic embedded models, hampers common learning lessons to draw from and build on. A Causal Loop Diagram (CLD), which is a visual representation of the dynamic relationships within a modelled system, is key to SD modelling, yet approximately a third of the studies (n = 6) did not include it in their publications. The CLDs are particularly informative in terms of understanding how economic aspects are positioned within dynamic relationships and how they influence them. Finally, a limited number of studies characterised the uncertainties of the economic analyses (n = 13) according to the CHEERS guidelines or reported on the statistical significance between policy scenarios in the overall SD models (n = 3) according to Rahmandad and Sterman’s guidelines. Characterising uncertainty enables decision-makers to better understand the information available, particularly in policy-making scenarios. While stakeholder input throughout the modelling process could arguably compensate for uncertainty, only five studies reported expert or stakeholder input and qualitative work as part of their SD approach. This is surprising, given expert or stakeholder engagement is a key feature of SD modelling and can help improve the predicting power of the model through assurance on whether the model is valid and representative of the real-world setting.

**Table 2:** Completeness of reporting of economic efficiency analysis and SD modelling in studies.

	Ahmad, 2005	Ahmad, 2005	Alirezaei et al., 2017	Al-Foraih et al, 2020	Assaf, 2009	Ansah et al., 2021	Chen, 2020	Duintjer Tebbens and Thompson, 2009	Erten et al., 2016	Evenden et al., 2005	Evenden et al., 2020	Hirsch et al., 2012	Hirsh et al., 2014	Homer et al., 2010	Honeycutt et al., 2019	Kivuti-Bitock et al., 2014	MacAskill et al., 2021	Macmillan et al., 2014	Mahmoudian-Dehkordi, et al., 2017	Schade and Rothengatter, 2005	Milstein et al., 2011	Shih and Tseng, 2013	Sluijs et al, 2021	Smith and Van Ackere, 2000	Tejada et al, 2013	Tengs et al., 2001	Tuulonen et al., 2009	Van Zyl et al, 2020	Yarnoff et al., 2019
**Quality criteria for reporting on SD modelling:**																													
Economic Analysis Methods	✓	✓	✓	✓	✓	✓	✓	✓	✓	✓	✓	✓	✓	✓	✓	✓	✓	✓	✓	✓	✓	✓	✓	✓	✓	✓	✓	✓	✓
Time horizon of analysis	✓	✓	✓	✓	✓	✓	✓	✓	✓	✓	✓	✓	✓	✓	✓	✓	✓	✓	✓	✓	✓	✓	✓	✓	✓	✓	✓	✓	✓
Assumptions	✓	✓		✓	✓	✓	✓	✓	✓	✓	✓	✓	✓	✓	✓	✓	✓	✓			✓	✓	✓	✓	✓	✓	✓	✓	✓
Study Parameters	✓	✓	✓	✓	✓	✓	✓	✓	✓	✓	✓	✓	✓	✓	✓	✓	✓	✓	✓	✓	✓	✓	✓	✓	✓	✓	✓	✓	✓
Characterising uncertainty										✓	✓		✓	✓	✓		✓	✓			✓	✓	✓		✓	✓	✓		
Limitations described						✓		✓	✓	✓	✓		✓	✓	✓	✓	✓	✓			✓	✓	✓		✓	✓	✓		✓
Cost-related conclusions	✓	✓		✓	✓	✓	✓	✓	✓	✓	✓	✓	✓	✓	✓	✓	✓	✓	✓	✓	✓	✓	✓	✓	✓	✓	✓	✓	✓
Unit of Analysis	✓	✓		✓	✓	✓	✓	✓	✓	✓	✓	✓	✓	✓	✓	✓	✓	✓	✓	✓	✓	✓	✓	✓	✓	✓	✓	✓	✓
Data sources	✓	✓	✓	✓	✓	✓	✓	✓	✓	✓	✓	✓	✓	✓	✓	✓	✓	✓	✓	✓	✓	✓	✓	✓	✓	✓	✓	✓	✓
**Quality criteria for reporting on economic analysis:**																													
Use of CLD			✓	✓	✓	✓	✓	✓	✓	✓	✓	✓		✓	✓	✓	✓	✓	✓	✓		✓	✓	✓	✓			✓	✓
Simulation Algorithm			✓	✓	✓	✓	✓	✓	✓	✓	✓				✓	✓	✓	✓	✓			✓	✓	✓	✓			✓	✓
Detailed description of steps	✓	✓	✓	✓	✓	✓	✓	✓	✓	✓	✓	✓	✓	✓	✓	✓	✓	✓	✓		✓	✓	✓	✓	✓	✓	✓	✓	✓
Software/hardware platforms	✓	✓	✓	✓	✓	✓	✓	✓	✓	✓	✓	✓	✓	✓	✓	✓	✓	✓	✓	✓	✓	✓	✓	✓	✓	✓	✓	✓	✓
Pre-processing steps	✓	✓	✓	✓	✓	✓	✓	✓	✓	✓	✓		✓	✓	✓	✓	✓	✓	✓		✓	✓	✓	✓	✓	✓		✓	✓
Parameter settings required	✓	✓	✓	✓	✓	✓	✓	✓	✓	✓	✓		✓	✓	✓	✓	✓	✓	✓		✓	✓	✓	✓	✓	✓		✓	✓
Iterations per scenario	✓	✓	✓	✓	✓	✓	✓	✓	✓	✓	✓		✓	✓	✓	✓	✓	✓	✓		✓	✓	✓	✓	✓	✓	✓	✓	✓
Post-processing steps	✓	✓	✓	✓	✓	✓	✓	✓	✓	✓	✓		✓	✓	✓	✓	✓	✓	✓		✓	✓	✓	✓	✓	✓		✓	✓
Statistical significance between scenarios			✓																			✓			✓				

The check-mark indicates the study reported against the criteria/guideline listed.

## 4. Discussion

### 4.1 Key findings

While SD modelling is increasingly being used to examine complex public sector challenges, it is unclear how the methodology has incorporated economic efficiency analyses, which provides crucial insight for policymakers about trade-offs in resource-allocation in their decision-making. This systematic review of published studies therefore examined the range and nature of economic efficiency analyses in SD studies to understand how cost dynamics have been evaluated from a systems perspective for public sector problems. The first objective of this review was to determine the policy target level (macro, meso, or micro level) for which the analysis has been conducted. All of the studies were situated at one of the macro, meso, or micro but none explored interactions between different levels. As a review by Currie et al. noted, this represents a missed opportunity as most complex problems cross boundaries between micro, meso, and macro, and are rarely addressed at only one level [65].

The second objective of this review was to compare approaches for how economic efficiency analyses have been incorporated with SD. This review has found that the combined use of SD with economic efficiency analysis to evaluate public sector innovations has been increasing, including in the health sector. The majority of the studies were conducted within the last decade of the review period, and almost a quarter (n = 7) within the past eighteen months, indicating that the need for the combined use of SD with economic efficiency analysis has been increasing. Economic efficiency analysis within SD broadly fell into two categories: as embedded sub-models or as cost calculations based on the outputs of the SD model. The limitations described in the studies, primarily regarding the assumptions or estimations, are consistent with most types of simulation models and are not necessarily specific to the practice of combining economic efficiency analyses with SD.

The final objective of this review was to evaluate the quality and completeness of reporting of the economic efficiency analyses and SD modelling. The CHEERS checklist and Rahmandad and Sterman’s guidelines measure the quality and completeness of reporting rather than that of the underlying research, but these aspects are still very important, particularly for an emerging methodological approach, for reproducibility, and for influencing policy [24, 25]. A recent systematic review of SD applications in health and medicine more broadly, noted considerable shortcomings in model documentation, calibration, and validation in included publications, which is confirmed in this review [17].

SD is an iterative approach to policy analysis and design that recognises the complexity of problems, and is greatly strengthened when expert or stakeholder perspectives are included in the process. This has particularly valuable potential in the health sector where a diverse set of stakeholders can be involved. Most of the studies did not report on such consultations, and this represents a missed opportunity to strengthen the validity and credibility with decision-makers of the recommendations. There was little information presented in the publications on whether these studies influenced actual decisions or policies, or how effective they were which may not be possible given the time-lag of evidence to policy, but we recommend SD practitioners more explicitly report on whether policymakers were involved in the process.

### 4.2. Implications for policy-influence

The overall paucity of studies from multidisciplinary teams, however, suggests its full potential is not being met to support decision-making processes across a range of public sectors and geographies, particularly health policy. Most notably, no SD studies were found that examined the allocative efficiency of policy options in a Covid-19 context, despite the significant impact of the pandemic on human health and government budgets. Jay Wright Forrester, considered the founding father of SD, stated that “the failure of system dynamics to penetrate governments lies directly with the system dynamics profession and not with those in government” [65]. It is important for the SD community to facilitate making its methodology more mainstream and disseminating its contributions across disciplines, as results could help allow public sector decision-makers limited deploy limited resources better. In the process, its application prompts organizations to ask “what if?” questions, which can reveal the unforeseen implications of innovations. This is part of what is considered the purpose of the ‘anticipation’ dimension of Stilgoe et al.’s (2013) Framework for Responsible Innovation, which aims for more responsibility in the governance of emerging science and innovation through four integrated dimensions: anticipation, reflexivity, inclusion, and responsiveness [66]. Responsible innovation acknowledges that innovations can be unpredictable in terms of impacts, beneficial or otherwise and the use of SD to test alternative scenarios in a systematic way and assess their costs and benefits, can help ensure more responsible practice in the introduction of innovations in the public sector.

## 5. Limitations

This study relies on English-language studies available in the public domain and the use of SD terminology in the title or abstract, and it is therefore likely that some studies using SD may have been missed that are not published or used different terminology to describe their modelling approach. Furthermore, there may be debate over our selection framework, particularly the exclusion of applications of economic efficiency analyses with SD in the private sector.

## 6. Conclusion

The Covid-19 pandemic has highlighted the role and obligations the world’s public sectors face in tackling major challenges. Decision-makers have to grapple not only with ensuring the cost-effectiveness of policy measures during disruptions, but also ensure the protection of key sectors such as the health system or R&D ecosystem. Systems science modelling approaches combined with economic allocative efficiency analysis can play an important role in producing realistic evidence on policy options for decision-makers. There is a significant lack however in the scientific literature, assessing the economic allocative implications of policies in complex systems. This is the first systematic review examining the range and nature of economic efficiency analyses with SD methodologies for complex public policy problems. This review reveals that SD has a high applicability and demonstrated capability to evaluate the economic efficiencies of public sector innovations but is currently not utilized to its full potential to help decision-makers in developing effective actions. Future modelling studies should adhere more closely to good practice guidelines, in particular uncertainty and statistical significance analysis. Further application of this approach in the health sector would be welcome to develop the methodology further.

## Supporting information

S1 Table Search terms and strategy. (DOCX)

S1 Appendix List of source articles in the systematic review. (DOCX)

S2 Table PRISMA checklist. (DOCX)
