# Supplementary material for: Using system dynamics modelling to assess the economic efficiency of innovations in the public sector - a systematic review
Source: PLoS One. 2022 Feb 10;17(2):e0263299. doi: 10.1371/journal.pone.0263299 (PMC8830692; doi:10.1371/journal.pone.0263299)
Supplement: S1 Table — (DOCX) [file pone.0263299.s001.docx]

**S1 Table: Search Terms and Strategy**

|  | Topic | Search Terms |
| --- | --- | --- |
| 1. | Economic evaluation | (Econom* OR cost* OR expenditure OR (economic analys*) OR (economic stud*) OR (economic model*) OR (economic evaluation*) OR (cost-benefit) OR (cost minimisation) OR (cost minimization) OR (cost-utility) OR (cost-effective*) OR financ*)  AND  Economics [MeSH] |
| 2. | System Dynamics | ((System* dynamics) OR ((system* thinking) AND model*))  AND  Systems Analysis [MeSH] |
| 3. | Policy | (policy or policies)  AND  Policy [MeSH] |

**Search Strategy:**

The search strategy for each database includes the following combinations: 1) AND 2) AND 3).
