# Supplementary material for: Using system dynamics modelling to assess the economic efficiency of innovations in the public sector - a systematic review
Source: PLoS One. 2022 Feb 10;17(2):e0263299. doi: 10.1371/journal.pone.0263299 (PMC8830692; doi:10.1371/journal.pone.0263299)
Supplement: S1 Appendix — (DOCX) [file pone.0263299.s002.docx]

**S1 Appendix: List of source articles in the systematic review**

1. Ahmad, S. 2005. See reference list for details.
2. Ahmad, S. 2009. See reference list for details.
3. Al-Foraih, R, Sreekanth KJ, Alasseri R, and Al-Osaimi S. 2020. See reference list for details.
4. Alirezaei, M. Onat, N., Tatari, O., and Abdel-Aty, M. 2017. See reference list for details.
5. Ansa JP, Sheng Hng KL, Ahmad S, Goh C. 2021. See reference list for details.
6. Assaf, H. 2009. See reference list for details.
7. Chen YC. 2020. See reference list for details.
8. Duintjer Tebbens and Thompson. 2009. See reference list for details.
9. Erten MZ, Fernandez LP, Ng HK, McKinnon WC, Heald B, Koliba CJ, Greenblatt MS. 2016. See reference list for details.
10. Evenden D, Harper PR, Brailsford SC, and Harindra V. 2005. See reference list for details.
11. Evenden D, Brailsford SC, Kipps CM, Roderick PJ, Walsh B. 2020. See reference list for details.
12. Hirsch GB, Edelstein BL, Frosh M, Anselmo T. 2012. See reference list for details.
13. Hirsch, G. Homer, J., Trogdon, J., Wile, K., and Orenstein, D. 2014. See reference list for details.
14. Homer J, Milstein B, Wile K, Trogdon J, Huang P, Labarthe D, Orenstein D. 2010. See reference list for details.
15. Honeycutt A, Bradley C, Khavjou O, Yarnoff B, Soler R, Orenstein D. 2019. See reference list for details.
16. Kivuti-Bitok, L., McDonnell, G., Abdul, R., Pokhariyal, G. 2014. See reference list for details.
17. MacAskill, S., Sahin, O., Stewart, RA., Roca, E., Liu, B. 2021. See reference list for details.
18. Macmillan, A. Connor, J., Witten, K., Kearns, R., Rees, D., and Woodward, A. 2014. See reference list for details.
19. Mahmoudian-Dehkordi, A. and Sadat, S. 2017. See reference list for details.
20. Milstein B, Homer J, Briss P, Burton D, Pechacek T. 2011. See reference list for details.
21. Schade, W. and Rothengatter, W. 2015. See reference list for details.
22. Shih, Y. and Tseng, CH. 2014. See reference list for details.
23. Sluijs, T., Lokkers, L., Ozsezen, S., Veldhuis, GA., Wortelboer, HM. 2021. See reference list for details.
24. Smith, P. and Ackere, A. 2002. See reference list for details.
25. Tejada, J., Ivy, J., King, R., Wilson, J., Ballan, M., Kay, M., Diehl, K., Yankaskas, B. 2014. See reference list for details.
26. Tengs TO, Osgood ND, Chen LL. 2001. See reference list for details.
27. Tuulonen, A., Salminen, H., Linna, M. 2009. See reference list for details.
28. Van Zyl, A. and Jooste JL. 2020. See reference list for details.
29. Yarnoff B, Bradley C, Honeycutt AA, Soler RE, Orenstein D. 2019. See reference list for details.
